# Hydrothermal Carbonization of Biomass for Electrochemical Energy Storage: Parameters, Mechanisms, Electrochemical Performance, and the Incorporation of Transition Metal Dichalcogenide Nanoparticles

**DOI:** 10.3390/polym16182633

**Published:** 2024-09-18

**Authors:** Manuel Prieto, Hangbo Yue, Nicolas Brun, Gary J. Ellis, Mohammed Naffakh, Peter S. Shuttleworth

**Affiliations:** 1 Instituto de Ciencia y Tecnología de Polímeros (ICTP-CSIC), Juan de la Cierva, 3, 28006 Madrid, Spain; manuel.pl@ictp.csic.es (M.P.); gary.ellis@csic.es (G.J.E.); 2 Escuela Técnica Superior de Ingenieros Industriales, Universidad Politécnica de Madrid (ETSII-UPM), José Gutiérrez Abascal, 2, 28006 Madrid, Spain; 3 Guangdong Provincial Key Laboratory of Plant Resources Biorefinery, School of Chemical Engineering and Light Industry, Guangdong University of Technology, Guangzhou 510006, China; hangbo.yue@gdut.edu.cn; 4 ICGM, Univ. Montpellier, CNRS, ENSCM, 34293 Montpellier, France; nicolas.brun@enscm.fr

**Keywords:** biomass, carbons, hydrothermal carbonization, supercapacitors, capacitance, transition metal dichalcogenides

## Abstract

Given the pressing climate and sustainability challenges, shifting industrial processes towards environmentally friendly practices is imperative. Among various strategies, the generation of green, flexible materials combined with efficient reutilization of biomass stands out. This review provides a comprehensive analysis of the hydrothermal carbonization (HTC) process as a sustainable approach for developing carbonaceous materials from biomass. Key parameters influencing hydrochar preparation are examined, along with the mechanisms governing hydrochar formation and pore development. Then, this review explores the application of hydrochars in supercapacitors, offering a novel comparative analysis of the electrochemical performance of various biomass-based electrodes, considering parameters such as capacitance, stability, and textural properties. Biomass-based hydrochars emerge as a promising alternative to traditional carbonaceous materials, with potential for further enhancement through the incorporation of extrinsic nanoparticles like graphene, carbon nanotubes, nanodiamonds and metal oxides. Of particular interest is the relatively unexplored use of transition metal dichalcogenides (TMDCs), with preliminary findings demonstrating highly competitive capacitances of up to 360 F/g when combined with hydrochars. This exceptional electrochemical performance, coupled with unique material properties, positions these biomass-based hydrochars interesting candidates to advance the energy industry towards a greener and more sustainable future.

## 1. Introduction

**Figure 1:**
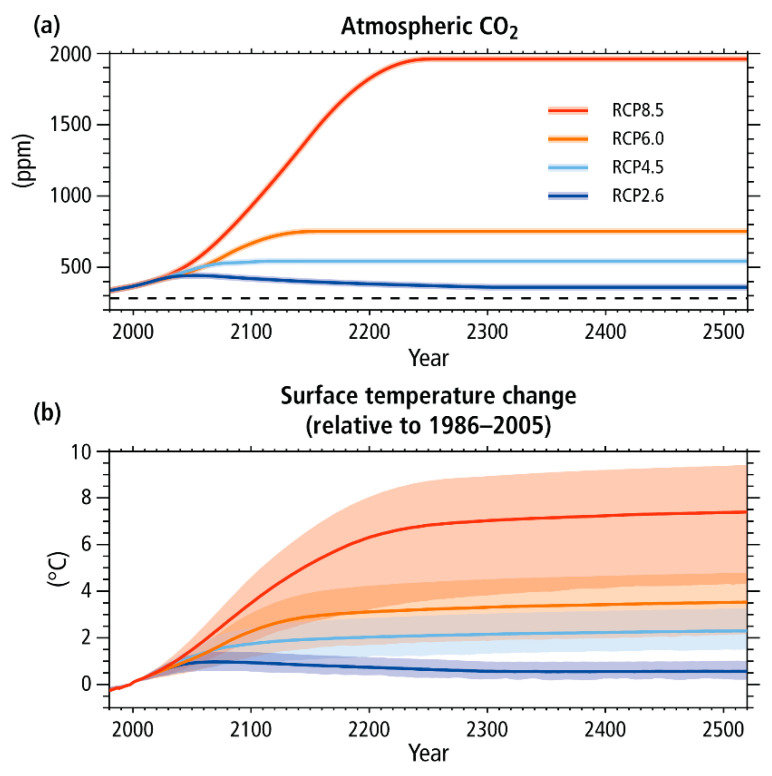
Temperature variation scenarios for the next 100 years [7], where RCP is ‘Representative Concentration Pathway’. (**a**) represents CO_2_ concentration scenarios and (**b**) represents global temperature increase scenarios. The dashed line indicates pre-industrial CO_2_ concentration.

The current global energy situation faces the challenge of transitioning away from fossil fuels to more sustainable alternatives in order to achieve the crucial goals outlined in the COP28 Paris agreement. These goals aim to limit global warming this century to below 1.5 °C [1]. To accomplish this, the energy sector, which is responsible for approximately two-thirds of all greenhouse gas (GHG) emissions, must significantly increase the implementation of renewable energy sources from 29% (2020) to approximately 60% by 2030 and 85% by 2050 [2]. These challenges are expected to worsen due to the projected increase in energy demand [3,4], an inevitable rise in energy prices [5], and the continuing growth of the world’s population [6]. Figure 1 illustrates several scenarios for global CO_2_ emissions, which are directly linked to an increase in global warming.

To mitigate global warming and achieve a negative net global emission, it is essential to replace fossil fuels with renewable alternatives. This transition requires intelligent and efficient energy use, along with the conservation of energy through advanced, state-of-the-art storage technologies. Estimates indicate that the current level of renewable energy must increase by an annual rate of 7% until 2030 to align with the Sustainable Development Scenario. Among renewable electricity-generating technologies, hydropower holds the largest share of the renewable energy sector, accounting for over 40% (1400 GW), followed by wind at 34% and solar at 25% at 34% and 25% [8]. Notably, in the broader spectrum of renewable energy (covering electricity, heat, transport, etc.), bioenergy in solid, liquid or gas form plays the most important role, contributing approximately five times more than the combined output of solar photovoltaics and wind.

Biomass, used to generate bioenergy derived from plants, their residues, food waste, and other sources, has gained prominence as a vast and widely available renewable resource, often regarded as carbon neutral. Within the total biosphere, approximately 550 gigatons of carbon (Gt C) are attributed to biomass, with the majority—450 Gt C—coming from plant sources [9]. Annually, agricultural production of lignocellulosic biomass in the EU28 is estimated at 419 Mtonnes of dry matter [10], with carbohydrates constituting 75% of this, primarily sourced from C5 and C6 pentose and hexose units. Only 5% of this biomass is used for food and non-food competitive purposes, alongside an additional 1.3 billion tonnes of food waste, presenting a substantial opportunity [11]. However, the utilization and conversion of these highly diverse feedstocks into useable products pose challenges due to significant variations in their chemical and physical properties. This process can also be costly as these materials typically exhibit low densities, low energy values, the presence of contaminants, and high moisture content, rendering many processing methodologies unsuitable [12]. 

One promising approach to addressing these challenges is wet torrefaction, commonly known as hydrothermal carbonization (HTC). HTC effectively converts mixed, wet biomass into carbonaceous materials with higher calorific value, resulting in a positive net energy yield [13]. When these HTC products are used as solid fuels, they can be considered more energetically efficient than conventional carbonization processes, depending on the plant’s geographical location and the targeted application. Additionally, there is significant potential for further optimization [14]. Notably, when HTC is employed as a pre-treatment for preparing carbon electrodes for energy storage applications, it is considered significantly more sustainable than traditional carbon materials [15].

The HTC process offers several advantages. It is applicable to any type of wet biomass, making it more energetically efficient than conventional carbonization processes [16,17]. Moreover, HTC prevents metal oxide contaminants—responsible for corrosion and fouling [18]—from remaining in the form of ash after combustion. Recently re-emerging as a valuable process, HTC efficiently transforms biomass within hours—into bio-oil, a gaseous fraction (mainly carbon dioxide) and, primarily, hydrochar, a solid, coal-like substance [19]—a transformation that takes nature millions of years to achieve. The concept of HTC dates back over a century and was first proposed by Friedrich Bergius in 1913, who later received the 1931 Nobel prize for his work on “chemical high-pressure methods” [20]. During HTC, the feedstock is heated in an aqueous medium at relatively low subcritical temperatures, typically between 150 and 250 °C, under self-generated pressures. At higher temperatures the process is considered hydrothermal liquefaction or hydrothermal vaporization [21].

**Figure 2:**
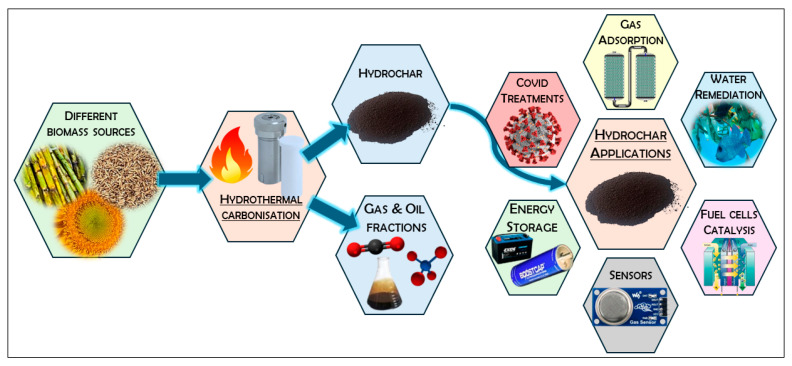
Schematic on hydrothermal carbonization precursors, products, and applications.

After HTC processing, the resulting hydrochar, similar to pyrochar produced through pyrolysis, finds applications in various fields [22], as depicted in Figure 2. It can be used as a soil fertility aid, enhancing water and nutrient retention in quick-draining soils, for GHGs sequestration [23,24,25], or as an adsorbent for wastewater remediation [26,27,28,29]. For instance, Li et al. demonstrated that hydrochar produced from rice straw exhibits significant adsorption efficiency for model heavy metals, dyes, antibiotics, and aromatic compounds due to its rich chemical functionality [30]. Hydrochars have also been employed to create carbon quantum dots for therapeutic treatment of HCoV-229E human coronavirus infection [31]. While hydrochars have diverse applications, their main focus has been in the energy sector. Co-firing hydrochar with coal as a ‘clean’ solid fuel replacement has gained attention due to the higher energy densities of hydrochar, ranging from 45% to 91% more than the original lignocellulosic feedstock, with final Higher Heating Values (HHVs) in the range of 24–30 MJ kg^−1^ [32]. The resulting solid not only boasts improved energy densities but also exhibits good aromaticity, with Van Krevelen H/C and O/C ratios comparable to lignite or coal [33,34,35]. It possesses good friability, allowing it to achieve particle sizes suitable for coal-fired power stations (≤74 μm for pseudo-fluid behavior [36]), it has low ash content, and is more hydrophobic, thus maintaining lower moisture content than the original feedstock [19]. 

In addition, HTC has proven to be a beneficial pre-treatment for pyrolysis or activation, leading to the synthesis of highly porous carbonaceous materials with increased mass and carbon yields and improved textural properties [37,38]. For this reason, HTC has become increasingly prevalent as a preliminary step for the preparation of highly porous carbon adsorbents tailored for gas adsorption/separation and wastewater treatment. Within the energy sector, hydrochars are undergoing extensive testing and are viewed as a more sustainable alternative to non-renewable carbons, particularly in the development of electrodes for fuel cells, batteries and electrochemical double-layer capacitors, commonly known as supercapacitors (SCs). However, it should be noted that these materials require further thermal treatment at higher temperature to reach adequate conductivity. 

The rapid growth in energy storage systems in the electronics industry over the last few decades has fueled research into more durable electrode materials. This growth is particularly evident in SCs, which complement batteries with excellent power densities, cycling stabilities and fast charge–discharge rates, though they have comparatively lower energy densities. These features make SCs suitable for short-duration power applications, such as uninterruptable power supplies, load levelling, LED devices, solar arrays, micro energy harvesting, and hybrid and electric vehicles equipped with regenerative braking and start–stop energy saving systems, among others [39]. This trend is reflected in the predicted Compound Annual Growth rate (CAGR) of 23% for the period of 2020–2027 [40]. In comparison, pumped hydro-storage, one of the most popular and widely used energy storage mechanisms (constituting 95% of utility-scale energy storage in the US) [41], has a CAGR of only 2% over the same period [42].

With the sustained demand on electrochemical storage devices, continuous efforts are being made to increase their energy densities without significantly affecting their power densities. Consequently, supercapacitor materials capable of undergoing Faradaic reactions have gained particular interest. One promising approach to enhance the electrochemical performance of HTC biomass is the incorporation of conductive nanoparticles (NPs) into the internal structure of the hydrochar. These can include carbon NPs including graphite, graphene, graphene oxide (GO), carbon nanotubes (CNTs), and nanodiamonds (NDs), transition metal oxides, such as iron (Fe_x_O_y_) [43], manganese (MnO_2_) [44], NiO [45], and mixed-metal oxides [46], amongst others. The reader is referred to recent reviews on the inclusion of such nanoparticles into biomass-derived carbons [47,48,49]. Other emerging nanoparticle types for electrochemical capacitor electrodes include metal-organic frameworks (MOFs) [50], newly developed MXenes [51], transition metal dichalcogenides (TMDCs), and their hybrids [52,53]. In this regard, we pay special attention to TMDCs that are of particular interest due to their potential for environmental applications and the innovative opportunities they present when incorporated with biomass-derived hydrochars in electrochemical energy storage devices. This novel incorporation enhances both conductivity and capacitance values of hydrochars, making them highly promising for energy storage applications. TMDC nanomaterials, which can be synthesized as 0D, 1D, and 2D structures, exhibit excellent mechanical and tribological properties. They have been utilized in polymer composites to improve wear resistance and crystallization behavior [54], as medical lubricants and coatings [55], for field emitting transistors [56] and for energy storage [57].

This review aims to provide a comprehensive overview of the literature on supercapacitor electrodes derived from activated hydrochar, as well as to explore the relatively uncharted territory of TMDCs mixed with hydrochars for energy storage applications. The focus is on activated hydrochars due to their cost-effectiveness and widespread industrial availability. Notably, other excellent reviews cover pore generation in hydrochars using methodologies such as soft and hard templating for various applications not limited to energy storage [58]. The first part of this review will delve into HTC processing, discussing the principal biomass composition, the mechanistic processes occurring in different biomass types during HTC, and key HTC process parameters. Following this, after this review will provide a basic overview of electrochemical energy storage processes, comparing various types, and then concentrates specifically on HTC hydrochars for energy storage. This includes details on activation routes, electrode material requirements, and an evaluation of capacitances reported in the literature for activated hydrochars derived from polysaccharides and lignin. The discussion will then extend to mixed biomasses. Finally, this review considers the relatively recent integration of TMDCs into biomass-derived carbons via hydrothermal methods for use in electrochemical energy storage, encompassing both batteries and supercapacitors.

## 2. Insights into the Hydrothermal Carbonization of Biomass

### 2.1. Lignocellulosic Biomass

The estimated available land on our planet is 13 × 10^9^ hectares, with only 37% of it utilized for agriculture [59]. This suggests that non-food competitive biomass can be sustainably sourced on most continents without competing with food crop production, provided proper management practices are implemented. Lignocellulosic biomass is composed of three primary components: cellulose, which constitutes 30–50% by weight [60], hemicellulose, which accounts for 15–35% by weight, and lignin. Additionally, 15–25% by weight comprises fats, proteins and inorganic materials. Furthermore, each inhabitant within the EU produces an average of approximately 130 kg of food waste (FW) per year (as of 2023) [61]. However, due to its high moisture content, FW is not suitable for direct pyrolysis, as it typically has lower heating values, falling below the required 3344 kJ/kg threshold [62]. If incinerated, it would result in significant greenhouse gas emissions. This issue can be mitigated using HTC or other suitable biorefinery techniques, adding value to this abundant waste at both the pre- and post-consumer stages. 

Cellulose, the most abundant biopolymer on Earth, is a linear chain β-(1→4) linked D-glucopyranosyl polysaccharide with a degree of polymerization ranging from a few hundred to ten-thousand, and exists in four crystal forms, although only types I_α_ and I_β_ occur naturally [63,64]. The stability and strength of cellulose derive from its linear structure and the ^4^C_1_ chair conformation of the glucopyranose units [65]. Following cellulose, hemicellulose is the second most abundant natural biopolymer. Hemicellulose consists of a variety of pentose and hexose sugar β-(1,4)-linked polysaccharides, which can be categorized into four key groups: β-glucans, xyloglucans, mannans and xylans, with the latter making up more than 30% of the total [12]. 

The third major component of lignocellulosic biomass, lignin, is an alkyl-aromatic biopolymer located within the plant cell wall. It is primarily composed of the three monolignols: coniferyl, p-coumaryl, and sinapyl alcohols. The composition of lignin can vary significantly depending on the plant species [66]. Lignin is also the most structurally complex component of lignocellulose, making its processing particularly challenging. As a result, the majority of lignin produced as a by-product in paper mills is simply burned for energy recovery, with only a small fraction being upgraded into value-added chemicals (e.g., aromatics) or advanced materials such as carbon fibers [67,68]. 

In addition to lignocellulosic material, which is more prevalent in pre-consumer waste with seed and stone removal, FW also contains other key components like lipids, proteins and carbohydrates, primarily in the form of starch. Some mono and disaccharides are also present, though in minor quantities [69]. Starch, the main energy storage carbohydrate in plants, contains of 97–99% α-glucans, specifically amylose and amylopectin, along with minor amounts of lipids, proteins, and other components. Its typical moisture content ranges from 10 to 20% [70]. Amylose, an isomeric polysaccharide of cellulose, is more flexible than amylopectin and contains approximately 1% α-(1→6) linked D-glucopyranosyl branch points. In contrast, amylopectin is more highly branched, with approximately 5–6% α-(1→4) glycosidic bonds.

### 2.2. HTC Mechanism and Relation to Biomass Type

The transformation of cellulosic biomass into a coal substitute dates back to the early work of Bergius [71]. This was followed by work in the 1920s on the HTC of other biomass types by Berl and Schmidt [72]. In fact, the origins of the HTC process can arguably be traced back even earlier, to the 1850s, with a patent by Vignoles for the ‘wet carbonization’ of peat. Despite these early developments, the basic HTC process has undergone significant changes over time. 

During HTC, various reactions can occur, largely depending on the type of biomass and the specific reactions conditions chosen. However, there is general agreement that the reaction mechanism typically involves several key steps: hydrolysis, which breaks down larger polymeric biomass into oligomers, dimers and monomers; dehydration, leading to the formation of furfural derivatives from the monosaccharides or oligosaccharides; decarboxylation; polymerization; and aromatization. 

The presence of subcritical water during hydrolysis plays a crucial role by reducing the activation energy required for bond cleavage. This allows the reaction to occur at more moderate temperatures, eliminating the need for strong acids to catalyze the process [73]. The subsequent reaction pathway depends on whether the produced sugar is a pentose (C5) or a hexose (C6). In the case of C5 sugars, they are thought to dehydrate into furfural, which can then be further converted into furfuryl alcohol using alcohols as hydrogen donors. This is followed by a hydrolytic ring-opening reaction that produces levulinic acid [74]. The remaining steps of the HTC process involve a series of reactions: (i) xylose undergoes a de Bruyn-van Ekenstein transformation to produce 1,2 ene-diol and D-xylulose; (ii) retro-aldol reactions lead to the formation of glyceraldehyde, which subsequently forms glycoaldehyde and formaldehyde; (iii) glyceraldehyde converts to dihydroxyacetone through another de Bruyn-van Ekenstein transformation; (iv) both glyceraldehyde and dihydroxyacetone dehydrate to form pyruvaldehyde; and (v) pyruvaldehyde undergoes hydration, resulting in acetic acid, formaldehyde, acetaldehyde, formic acid, and lactic acid [75].

In the case of C6 sugars, these molecules undergo dehydration, producing both cyclic and linear intermediate products. Some studies suggest that the linear intermediates can directly form humins [76], though in negligible amounts. The process ultimately leads to the formation of 5-hydroxymethylfurfural (HMF). Previous research indicates that the conversion of glucose and fructose to HMF (and furfural) occurs through the de Bruyn-van Ekenstein transformation, involving epimerization to fructose or mannose [77]. Prolonged exposure of HMF to the aqueous medium triggers a rehydration process that results in the formation of levulinic acid and formic acid [78]. This rehydration lowers the pH of the medium, further catalyzing dehydration and subsequent polymerization of HMF. 

The HMF pathway is believed to involve a series of reactions with 2,5-dioxo-6-hydroxy hexanal (DHH), leading to the formation of an initial dimer that further increases with additional HMF. DHH is hypothesized to result from the acid-catalyzed hydrolysis ring-opening of HMF. However, its detection in practice remains elusive, suggesting that it is a highly reactive intermediate. Caution should be exercised when accepting this assumed mechanism due to the lack of physical evidence. 

**Figure 3:**
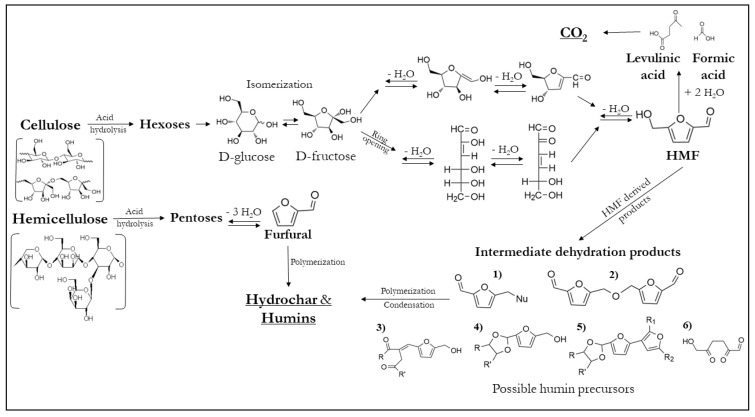
Mechanistic routes of cellulose and hemicellulose breakdown under hydrothermal conditions. Scheme constructed from information in references [76,77,80].

The process leading to the production of HMF is generally well-understood. However, the subsequent formation of humins is more difficult to comprehend due to the complexity and number of reactions involved. Humins can be categorized into two classes based on the reactive groups involved: the hydroxymethyl group and the formyl group, which lead to the formation of humin precursors, as shown in Figure 3. 

In the first scenario, the hydroxymethyl group can undergo nucleophilic substitution, acting as a leaving group (see Intermediate dehydration products; Product 1), or it can participate in etherification reactions (Product 2). On the other hand, the formyl group can engage in various reactions, such as aldolic condensation, typically with diketones at the α position, resulting in Product 3, as illustrated in Figure 3. Additionally, acetalization can occur in the presence of alcohols in the medium, leading to the formation of Product 4 [77]. From Product 4, dissociation of the hydroxyl group can produce highly reactive carbocations, which can initiate electrophilic substitution reactions, ultimately resulting in Product 5.

However, HMF is prone to undergoing additional reactions, including the aforementioned rehydration process that produces levulinic and formic acids, as well as the formation of 2,5-dioxo-6-hydroxyhexanal [79] (Product 6) and Diels–Alder reactions. The combination of these reactions contributes to the formation of humins, which are believed to comprise of approximately 60% furan rings and 20% ether or acetal linkers [80].

**Figure 4:**
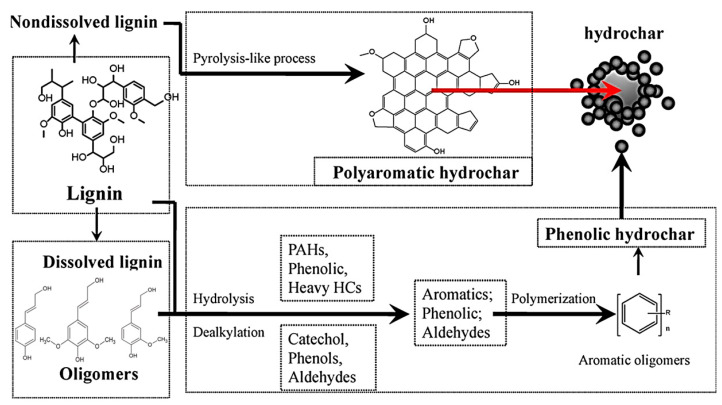
Lignin hydrothermal mechanism. Adapted from [19] with permission from Elsevier.

Lignin, the other major constituent of lignocellulosic biomass alongside cellulose and hemicellulose, has a more complex molecular structure. The mechanism governing lignin transformation differs significantly from that of the other materials, as illustrated in Figure 4. The process begins with the degradation of lignin into soluble fragments. The first step involves the dealkylation and hydrolysis of dissolved lignin into methoxy phenolics, which are subsequently converted into phenolic compounds. This stage, which competes with demethoxylation, alkylation and condensation reactions [81], is facilitated by the relative ease of cleaving C-O-C bonds compared to C-C bonds. Following this initial stage, these intermediates undergo cross-linking reactions, eventually leading to their repolymerization into hydrochar. Meanwhile, the undissolved fraction of lignin follows a pathway similar to pyrolysis, resulting in a polyaromatic hydrochar structure [19]. This dual pathway for lignin decomposition reflects its intricate nature and highlights the diverse mechanisms involved in its conversion into hydrochar. For a more detailed exploration of lignin types, chemistry, and underlying reactions, the authors refer readers to the following book chapter [81].

### 2.3. Parameters Governing the HTC Process

#### 2.3.1. Temperature

Temperature is arguably the most critical parameter in the HTC process, as it plays a key role in breaking the bonds within the feedstock. Its primary function is to provide the disintegration heat necessary to break the intermolecular links between the polymers. Additionally, temperature also influences the properties of water, which drives the reaction. In the previously mentioned ionic reactions intrinsic to this process, an increase in temperature reduces water viscosity, thereby enhancing the degree of feedstock degradation. This effect is attributed to the increased penetrative ability of water [82], and a decrease in the solvent’s polarity, which facilitates the dissolution of organic components [73]. If the temperature is too low to sufficiently disrupt the biomass structure, a pyrolysis process can take place as a response opposed to the reactions of the monomers in the homogeneous reaction [19]. However, excessively high temperatures are not advisable as they may promote the formation of secondary char through polymerization, which could dominate the mechanisms underlying hydrochar formation [83]. 

Several studies have demonstrated a direct correlation between an increase in temperature and a decrease in solid yield during HTC, accompanied by an increase in gas concentrations (e.g., CO_2_, CH_4_, H_2_) due to dehydration, decarboxylation, and a reduction in volatile matter. Although higher temperatures often result in a lower yield, there is a notable increase in the High Heating Value (HHV) of the hydrochar. While optimizing both HHV and solid yield is desirable, HHV is not a crucial factor in electrochemical applications. Therefore, it is recommended to operate at relatively low temperatures in order to maximize solid yield, even if this results in a lower HHV. For example, in Lee’s studies [84] using *Chlorella vulgaris*, a decrease was observed in solid yield when increasing the temperature from 180 °C to 240 °C, with a peak in HHV at 200 °C. Similar trends have been reported in studies involving diverse materials such as cellulose [84], lignin [84], xylane [84], bamboo [85], nut shells [86] or small plants like *Hummus lupulus*, *Plumeria alba*, *Calophyllum inophyllum* [87], as well as tobacco stalk [88].

A general observation in the HTC process is that a temperature range of 150–230 °C appears optimal for maximizing solid production, while a range of 250–350 °C is ideal for liquid generation. At even higher temperatures, gas becomes the predominant product. These temperature ranges appear to be consistent across various materials, including cellulose [89,90], where the maximum solid yield occurs approximately 200 °C. Similar trends are observed in sugarcane bagasse, nut shells [86,91], peat [92], and other materials with diverse origins, such as poultry wastes [93], with optimum yields near 170 °C, and a slightly lower optimum temperature of 150 °C in the case of different types of algae [94].

Beyond yield optimization, temperature significantly influences the characteristics of the synthesized material. As temperature increases, the carbon content in the material rises, while hydrogen and oxygen contents decrease markedly [89]. Additionally, the degree of aromaticity has also been reported to increase with temperature [90,95], likely due to the distribution of hydrochar and a reduction in the number of reactive sites within the aromatic structures.

#### 2.3.2. Residence Time

Another important factor in the HTC process is residence time, as it significantly influences the severity of the reaction, although to a lesser extent than temperature when it comes to producing solid products. HTC is recognized as a slow process, with residence times ranging from a few minutes to several days. The impact of residence time is particularly pronounced in hydrolysis reactions up to a certain time point, beyond which its influence diminishes considerably [96,97].

The formation of secondary hydrocarbons (e.g., furfural) is strongly dependent on residence time, due to the polymerization durations required by these compounds [32]. In contrast, monomer formation is more affected by temperature than by residence time. It has been demonstrated that residence time controls the degree of feedstock decomposition, influencing both the hydrolysis and polymerization of monomers. This, in turn, affects the textural properties and particle sizes of the resulting hydrochars [88,89].

Effective biomass decomposition generally requires relatively short residence times, since hydrolysis and degradation are relatively fast reactions. Gao’s studies on cellulose [90] indicate that shorter residence times favor the hydrolysis of cellulose into water soluble materials,, inhibiting their further decomposition into heavy oils. As residence time increases, the solid yield decreases due to the rupture reaction that occurs in the first hydrothermal product of the heavy oil. Similar findings to those on cellulose have been observed in other materials, such as tobacco plants [88], where the solid yield decreases from 62% to 41% when residence time is extended from 1 to 12 h at 260 °C. A comparable trend is seen in peat [92], where yield drops from 85% to 65% when residence time is increased from 1 h to 8 h at 190 °C. However, this effect is less pronounced in materials like corn cobs [97], which show only a slight yield variation of approximately 1% when residence time is extended from 1 to 6 h at 250 °C. 

In summary, these studies suggest that longer residence times favor bio-oil production, particularly at low temperatures (approximately 150 °C). Additionally, as the temperature increases to approximately 250 °C, gas production also increases [90]. 

Residence time not only influences the concentration of products within the process but also affects their properties, as some experiments show. Processes with shorter residence times tend to produce products with higher HHV [97,98], largely due to the removal of oxygen from the biomass or the hydrolysis of hemicellulose. Some authors also suggest that longer residence times enhance the textural properties of the products after activation, improving porosity, pore volume, and BET surface area [99]. However, in some cases, these properties reach their optimal values at intermediate residence times (1–4 h), after which they begin to decline [88].

#### 2.3.3. Feedwater Acidity and Catalyst

Several studies have highlighted the significance of pH as a crucial parameter in the HTC process, as it plays a fundamental role in the production of organic acids during carbonization. These acids are essential intermediates that catalyze the decomposition of biomacromolecules and the formation of hydrochar. HTC is considered an autocatalytic process, where acids such as formic, acetic, lactic and levulinic are generated, leading to a reduction in pH [100]. Nevertheless, acids or bases can also be added as catalysts to increase the ionic strength of the medium, thereby accelerating the reaction or directing it towards the formation of the desired hydrochar [101]. 

In Yang’s study on nut shells [86], the solid yield was analyzed at pH values ranging from 4 to 13. The results revealed that the yield remained relatively constant between pH 4 and 10, but dropped significantly from 60% to 20% when the pH reached 13, indicating an increase in the generation of water-soluble products, suggesting liquefaction of the feedstock at elevated pH values. Similarly, studies on wheat straw [102], showed that within a pH range of 2 to 12, the solid yield remained stable at both 200 °C and 260 °C. Elemental analysis revealed that cellulose and hemicellulose were less reactive in basic pH conditions, in contrast to lignin. Similar trends were observed in studies involving sewage sludges [103,104] where solid yield varied by less than 5% across a pH range of 2 to 12, and in sawdust [105], which showed similar results at 250 °C, though yield variation reached 10% at 200 °C. Generally, hydrothermal treatment of feedstock in high pH feedwater contributes to the development of materials that, after activation, exhibit better surface area and pore volume in the hydrochar, although with a smaller pore diameter [102].

Alternatively, the use of small amounts of catalyst can enhance the degree of hydrolysis, although different catalysts are required depending on the specific hydrolysis reaction. Acid catalysts, such as sulfuric acid [106], are generally more effective for hydrolysis, while basic catalysts, such as RbOH or CsOH [107], promote bio-oil formation [99]. Another advantage of using catalysts is their ability to reduce NOx emissions, as these compounds are converted into nitrogen and water [99]. The properties of the catalysts play a crucial role in the reaction, emphasizing the important need for thermally stable, efficient, and cost-effective catalysts that exhibit high selectivity towards the desired product and yield.

#### 2.3.4. Feedstock

The structure and composition of the different types of biomass vary widely, influenced by factors such as the environment in which the feedstock has grown and the season. Moreover, each component of biomass responds differently to temperature variations. For instance, materials with higher cellulose and hemicellulose content tend to primarily produce bio oil [108], whereas feedstocks rich in lignin predominantly yield char [109]. This is due to the branched structure of lignin, which makes it more resistant to degradation [35,86,87]. The cellulose and lignin content in biomass can vary significantly, but in most agricultural wastes, it typically consists of approximately 40–50% cellulose, 25–30% hemicellulose, and 10–20% lignin [110,111,112]. However, there are instances where biomass contains as little as 9% or as much as 90% cellulose, more than 80% hemicellulose, and over 40% lignin [113,114,115]. Therefore, selecting the appropriate type of biomass becomes an important issue depending on the desired end product.

The HTC mechanism involves processes such as decarboxylation, dehydration, condensation polymerization, hydrolysis, and aromatization. Understanding these mechanisms depends on the type of biomass feedstock used, which plays a crucial role in the development of porosity during the activation of the material following the HTC process. To date, there are limited results linking feedstock concentration to the outcomes of the hydrothermal process. It is hypothesized that high concentrations of dissolved substrate might promote rapid polymerization of soluble substances, leading to the formation of larger spherical particles [19]. However, further research is needed to draw more definitive conclusions in this area.

#### 2.3.5. Heating Rate

Another important factor in the HTC process is heating rate, which generally does not promote hydrochar formation when reaching high values. Depending on the desired end product, high heating rates (10–20 K/min) are often employed to minimize residence time for secondary reactions leading to an increase in bio-oil yield and a corresponding decrease in solid production [19]. Studies on rapeseed, where the heating rate varied from 5 to 50 °C/min, demonstrated that higher heating rates resulted in a greater mass loss [116], a trend also observed in wood chips [117]. These findings suggest that lower heating rates lead to a higher degree of carbonization of the raw material, thereby achieving a higher solid yield.

#### 2.3.6. Pressure

Pressure plays a crucial role in the HTC process, primarily because it regulates both the decomposition rate and hydrolysis, especially when maintained above the critical pressure of the medium [99]. Controlling pressure is essential for directing reaction pathways towards the desired product, since pressure changes are closely linked with temperature variations, which, as mentioned earlier, significantly influence the final product yield.

Furthermore, it is well-established that high-density solvents can accelerate biomass breakdown [118], as they promote solvolysis, hydration and pyrolysis reactions during liquefaction, thereby facilitating the biomasses degradation. Achieving these higher solvent densities is possible through the application of higher pressures.

### 2.4. Hydrochar Pore Formation

HTC of saccharides and/or lignocellulosic materials typically results in the formation of hydrochars composed of aggregated, non-porous microspheres. To enhance the textural properties of hydrochars, i.e., increasing specific surface area and to finely tuning pore size to improve their electrochemical performance through enhanced adsorption capacity, dedicated synthetic approaches are necessary. In the literature, two main approaches have been employed to generate porosity within the internal structure of hydrochars: (i) templating approaches, where sacrificial templates are added in the initial hydrothermal reaction medium and later removed; and (ii) activation processes, where hydrochars undergo a thermal post-treatment in the presence of an activating agent.

Templating methods, used since the 1980s to produce mesoporous carbons, can be divided into two categories: hard templating and soft templating. Hard templating typically involves preparing a silica template with the desired porous structure, adding a carbon precursor, undergoing carbonization, and finally dissolving the silica framework [119]. In contrast, soft templating involves creating a porous structure using different agents, such as solvents or surfactants. The application of soft templates in HTC-derived carbons has gained prominence in recent years, leading to the production of micro and mesoporous carbons using different PEG-PPG block copolymer surfactants [120], and various renewable precursors such as xylose [121], fructose [122], and glucose [123].

The second approach, activation for porosity generation, includes both physical and chemical activation. In these methods, the material is exposed to moderate-to-high temperatures in the presence of an activating agent in order to develop an internal porous structure.

#### 2.4.1. Physical Activation

In physical activation, the precursor is exposed to a flow of steam or carbon dioxide, or both [124], at temperatures starting from 700 °C [21]. CO_2_ activation involves a C-CO_2_ reaction that leads to the removal of C atoms, opens closed pores, and widens existing pores [125]. In contrast, steam activation facilitates the release of volatile gases with partial devolatilization and results in the formation of a crystalline carbon structure [126].

Various authors have examined the impact of parameters such as, temperature, residence time, base material, and activating agent on the final porosity of physically activated, hydrothermally treated biomass. Antero et al. [127] prepared carbons from *Magonia pubescens* at temperatures ranging from 170 to 190 °C followed by steam activation at 700 °C. They observed that the carbon content (C%) decreased with increasing temperature, similarly to burn-off, and was inversely proportional to yield. This was attributed to a “less organized initial structure of the material and (its) lower thermal resistance” [127]. The pore size distribution showed two contributions centered at 3 and 30 nm that, coupled with pore volume data, indicated that the samples were mainly mesoporous with an important contribution from micropore. The largest surface areas were observed in samples prepared at 180 and 170 °C, with values of 441 and 360 m^2^/g, respectively. A similar approach was employed by Liu [128] using pinewood sawdust and rice husk. These materials were hydrothermally treated at 300 °C for 20 min, followed by CO_2_ activation at 800 °C for varying activation times (between 30 and 120 min). For both materials, carbon yield decreased with activation time, as did the micropore volume to total pore volume ratio (dropping from approximately 90% to 82%). This confirmed that all samples were predominantly microporous. However, the BET surface area increased with activation time, which the authors attributed to “the predominant role of micropore combination (collapse of the pore structure) rather than the micropores formation” [128]. In another study, Román [26] compared hydrothermally treated carbons derived from sunflower stems, walnut shells and olive stones: These were processed at 220 °C for 20 h and physically activated with carbon dioxide (at 800 °C) and air (at 250 °C) for 30 min. The results varied by material. For walnut shells and olive stones, the CO_2_ activation process led to lower burn-off, whereas for sunflower stems the opposite occurred. Despite these differences, the textural properties (BET area, micropore volume, and mesopore volume) followed the same trend, with higher values of BET and micropore volume (and consequently smaller mesopore volume in all samples except sunflower stem) when CO_2_ was used as the activating agent. Notably, regardless of the activating agent, all samples remained highly microporous, exhibiting type I isotherms characteristic of microporous materials with narrow micropores (typically less than 1 nm in width) [129].

#### 2.4.2. Chemical Activation

Chemical activation, on the other hand, involves placing the carbonaceous precursor in contact with a chemical activating agent, such as potassium hydroxide (KOH), sodium hydroxide (NaOH), metal salts, or acids. The mixture is then heated to moderately high temperatures, typically between 600 and 800 °C [21], followed by treatment with water and/or acids to neutralize the pH and remove residual potassium, sodium, or metal salts. After washing, the final material exhibits a porous structure and a higher surface area, achieved through a rapid, one-step process. However, a potential drawback is the risk of secondary environmental pollution during disposal from certain agents, such as zinc salts or phosphoric acid [130].

There is extensive literature on the use of chemical activation to enhance the textural properties of carbonaceous materials, with a significant focus on the chemical treatment of hydrochars. A notable example is Wang’s experiment [131] with hemp fibers. After HTC using an acidic solvent instead of water (at 180 °C for 24 h), the material was mixed with KOH in a 1:1 mass ratio and heated at 700–800 °C for 1 h. These materials exhibited approximately 50% microporosity of the total pore volume and surface areas above 1500 m^2^/g, with the material prepared at 750 °C showing a BET area of 2287 m^2^/g, being that with more micropores, while the sample prepared at 800 °C exhibited the highest mesopore contribution. All samples displayed type I/IV isotherms, and mesopore volume increased with the activation temperature. Other studies have focused on pure biomass components, such as cellulose and starch. For instance, Wei’s work [132] cellulose, starch, and wood sawdust were hydrothermal carbonized at 230–250 °C for 2 h. The resulting product was mixed with KOH in a 4:1 (KOH: Carbon) mass ratio and heated to 700–800 °C at a rate of 3 °C/min. The textural properties results showed specific surface areas exceeding 2000 m^2^/g, with some samples surpassing 2900 m^2^/g. These samples were predominantly microporous, with microporosity ranging from 75 to 90%. This type of activation has also been applied to mixtures of different materials, such as polysaccharides and algae. In Sevilla’s study [133], glucose and *Spirulina platensis* were hydrothermally processed at 180 °C for 24 h, then mixed with KOH in a 2:1 KOH/hydrochar mass ratio and heated at 650–750 °C. The results showed increases in BET area, micropore area and volume, total pore volume, and average micropore width with increasing activation temperature, although N and O percentages decreased due to their removal during the activation process. In all cases, microporosity exceeded 90% of the total pore volume, BET areas ranging from 1800 to 2200 m^2^/g. Comparing these studies, it is evident that increasing KOH content significantly influences micropore generation.

#### 2.4.3. Chemical or Physical Activation: Which Is Better When Targeting EDLC?

At this juncture, it is important to emphasize that the primary focus of this review is on the electrochemical application of HTC-treated biomass (and its modification with TMDCs), where the porosity of the carbonaceous electrodes plays a crucial role. The various types of electrolytes employed in supercapacitors have different molecular sizes, making it essential to tailor the electrode porosity to the specific electrolyte used, for optimal electrochemical performance. This explains why impressive capacitance values (>200 F/g in two-electrode capacitors) have been observed across different types of biomass-derived, hydrothermally treated electrodes, whether mesoporous [134], microporous [135], or hierarchically structured with combined micromesoporous features [136]. Notably, some studies have even reported increased capacitance when the pore size is less than 1 nm [137].

Within this context, comparing the chemical and physical activation process for the same material can provide insights into the porosity of the resulting materials. For example, Miliotti’s work [138] on HTC-treated lignin (200/270 °C; 2/4 h) compared the results of physical (CO_2_, 550 °C) and chemical (KOH, 600 °C) activation. Across all samples, the highest yield was observed at maximum temperatures and residence times, with chemical activation producing slightly higher yields than physical activation (33.1% vs. 30.5%). However, in terms of porosity, lower HTC temperatures (200 °C) appeared optimal for both activation methods. CO_2_ activation achieved higher porosity with a 2 h residence time, while chemical activation performed better with a 4 h period, resulting in BET areas of approximately 600 m^2^/g. Regardless of the parameters used, all samples exhibited type I (and II for KOH-activated) adsorption isotherms, indicating a predominantly microporous structure. In physical activation, micropore volume accounted for over 80% of the total pore volume, while in chemical activation, it was over 60%.

#### 2.4.4. Why Is HTC a Beneficial Pre-Treatment to Activation?

It is important to emphasize the advantages of HTC in preparing hydrochars for subsequent processes. HTC is recognized as an efficient method for pre-treating samples before pyrolysis [15,17] or activation [132]. Its ability to process wet biomass sets it apart from conventional heating methods, as it eliminates the need for a costly, energy-intensive drying stage. Additionally, hydrochars produced through HTC exhibit a hydrophobic nature, significantly enhancing dewatering efficiency. This large reduction in the moisture content lowers the thermal drying requirements, leading to energy savings. Moreover, HTC effectively removes metal oxides, such as potassium oxide and others from biomass, by dissolving them in water, which helps avoid corrosion and fouling problems –challenges that are more difficult to address using conventional heating methods [73].

At this stage, this review will focus primarily on the chemical activation of the hydrochar as it is a widely used process with demonstrated capability to develop carbonaceous materials with exceptional textural properties. Chemical activation also allows for good control of pore size [139] and achieves higher mass yield compared to physical activation [138,140], as well as good adsorption capacity, which is crucial for superior electrochemical performance.

## 3. Electrochemical Energy Storage—EDLCs

### 3.1. Overview

Within the realm of electrochemical energy storage, batteries and supercapacitors stand out as the primary devices. Batteries operate based on redox processes that convert chemical energy into electrical energy, allowing them to store large amounts of energy compared to electrochemical capacitors (commonly known as supercapacitors, SCs). However, batteries typically have longer charge/discharge cycles and a more limited lifespan than SCs [39]. In contrast, SCs are garnering significant research attention due to their superior power density, faster discharge time, higher efficiency and longer life cycle compared to batteries [141]. 

It is important to note that many materials currently used for electrode production originate from fossil sources, including carbon nanotubes (CNTs) [142,143,144], graphene [145,146,147] or conductive polymers [148,149]. However, there is a growing interest in materials from different origins, such as metal oxides [150,151] and their hybrid blends with the aforementioned materials [152,153]). Additionally, activated carbons (AC) derived from renewable sources are being explored for use in these applications. In this respect, hydrothermally treated carbonaceous materials, specifically hydrochars, are emerging as promising options for electrodes in SCs. 

**Figure 5:**
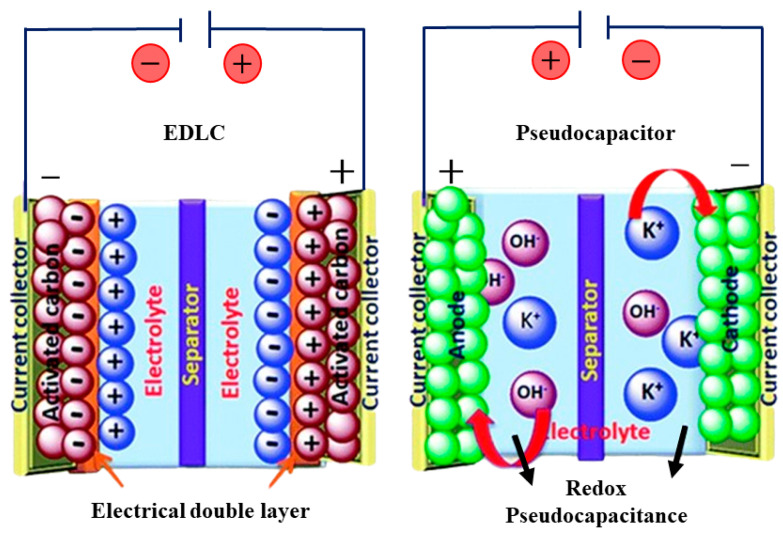
Energy storage mechanisms in a supercapacitor (adapted from [159]).

Electrochemical capacitors use two primary energy storage mechanisms, illustrated in Figure 5. The first, electrical double-layer capacitance (EDLC), involves energy storage through surface polarization of the electrode material. This occurs at the interface between the electrode and electrolyte, behaving like an electrostatic capacitor with a nanometer-scale dielectric known as the Helmholtz layer. In this mechanism, a potential difference is established between the electrode and electrolyte, creating a capacitance related to this potential and the charge density of electrons and ions at the electrode–electrolyte interface [154]. The second mechanism is pseudocapacitance, where energy storage is driven by oxidation-reduction reactions. This process is similar to battery energy storage but with minimal activation overpotential [154]. In pseudocapacitance, electrolyte ions move to oppositely charged electrode upon the application of voltage. Anions undergo oxidation, providing electrons to the circuit, while cations participate in electrochemical reduction reactions. Traditionally, pseudocapacitors have utilized materials such as transition metal oxides [155] and conducting polymers [156] for their electrodes. Additionally carbonaceous gels have shown potential in this role, due to the pseudocapacitive contributions of heteroatoms, such as oxygen (O), nitrogen (N) and sulfur (S) [157,158]. 

### 3.2. Electrode Properties

Sustainable carbonaceous materials sourced from renewables are emerging as promising candidates for electrode materials. Their appeal lies in their high surface area and porosity, coupled with the benefits of low manufacturing costs [160]. However, achieving optimal electrochemical performance with these materials requires careful consideration of several key parameters to ensure that their full potential is harnessed effectively.

The first critical parameter in electrode material design is porosity, which significantly affects the surface area through the tunability of the pores during the activation process. A key challenge lies in achieving a balance between enhancing pore size and maintaining electrical conductivity, as these parameters are inversely related [160]. Optimal energy and power density in electrodes require a combination of micropores and mesopores. Micropores, with their high surface area-to-volume ratio, contribute substantially to surface area, play a crucial role in selective adsorption process [161], and partly determine the electrode’s capacitance [162,163]. In contrast, mesopores not only contribute to the surface area but also facilitate ion diffusion due to their larger size and greater accessibility [161,164], significantly enhancing capacitance, especially at high currents [165]. 

Regarding porosity, in addition to pore size distribution, inter-pore connectivity is also important. Enhanced connectivity facilitates ion transport, directly effecting total capacitance [164]. Synthesizing mesoporous materials with optimal pore spacing for effective electrolyte diffusion remains a challenge [166]. A common approach to obtain materials with hierarchical porous structures involves the use of templates [163,167,168], but recent advancements are exploring alternatives to conventional templates [169,170]. Certain types of biomass in this regard can be particularly favorable, owing to their inherent, multichannel structure with interconnected pores [171].

The presence of functional groups is another factor to consider. Biomass, with its high oxygen and nitrogen content, promotes both pseudocapacitance and conductivity through its functional groups [172]. These groups are also responsible for pore wettability, which is crucial for ion penetration and transport. The presence of heteroatoms like oxygen, nitrogen, and boron is sought after the synthesis of electrodes, as they improve the wettability and electrical conductivity of the carbonaceous matrix [173]. However, careful control over the number of oxygenated functional groups is necessary, as over-oxidation can detrimentally affect the material’s porosity [166].

### 3.3. Biomass-Derived Electrode Materials for Supercapacitor Applications Prepared without HTC-Pretreatment

Many authors have presented interesting results synthesizing biomass-derived electrodes through processes other than HTC. Some of these results, for both aforementioned energy storage mechanisms, have been summarized in Table 1.

Several conclusions on the key factors that influence performance can be drawn from this table. Firstly, the choice of electrolyte is critical. Ionic electrolytes typically result in lower capacitances due to larger ion sizes. This is exemplified in the use of glucose-derived AC [174], which, despite having excellent textural properties, achieves a capacitance of only 175 F/g—considerably lower than other carbons using aqueous electrolytes. However, the advantage of ionic liquids is their ability to operate at higher potential windows, yielding higher energy and power densities.

Additionally, the effect of pseudocapacitance in enhancing capacitance of carbon materials, is notable. For example, Li’s study [175] on ginger straw carbon demonstrates this effect, where oxygenated and nitrogenated groups contribute to increased capacitance, as evidenced by voltammogram curves. A similar observation is made in Huo’s work [176] with nitrogen-doped carbon nanosheets derived from silk.

Furthermore, comparing the electrochemical performance of all materials in a two-electrode system, the textural properties emerge as crucial. A high specific surface area with appropriately sized pores to accommodate the electrolyte’s ions, is essential. For example, AC derived from paulownia flowers [177], with an optimal pore size distribution peaking at 2 nm (while the electrolyte ion size is less than 1 nm), facilitates electrolyte ion access and efficient diffusion, achieving a capacitance of nearly 300 F/g.

**Table 1:** Biomass derived electrodes (NOT hydrothermally pre-treated).

Electrode Material	Synthesis Method	Electrolyte	Stability	Microporosity (%)	BET Area (m^2^/g)	Cap (F/g) ^1^
AC from paulownia flower [177]	Pyrolysis at 600 °C, mix with KOH (3:1 KOH: carbon ratio) and carbonization at 800 °C	1 M H_2_SO_4_	93% retention after 1000 cycles	81%	1159	297
AC from wheat straw [178]	Pyrolysis at 800 °C, KOH (5:1) activ. ** at 800 °C	PVA/KOH	97.6% after 5000 cycles	62%	2115	294/296 *
Ginger straw-based AC [175]	Carbonization at 700 °C	6 M KOH	88% after 6000 cycles	65%	720	243
AC from rice husk [179]	Mix with ZnCl_2_ (4:1) followed by microwave heating (600 W).	6 M KOH	28% at 20 A/g	15%	1565	240
Carbon nanosheets derived from silk [176]	Mix with ZnCl_2_ (2.5:1) followed by annealing at 900 °C	EMIMBF_4_	92% after 10,000 cycles	18%	2494	213
Porous carbon from tissue papers [180]	KOH (2.5:1) activ. at 700 °C	6 M KOH	58 F/g at 100 mV/s	Mainly microporous	1320	200 (at 1 mV/s)
AC from biomass waste [181]	Pyrolysis at 500 °C and KOH (3:1) activ. at 700 °C	6 M KOH	75% at 10 A/g	85%	1831	197/289 *
AC from peanut shell [179]	Mix with ZnCl_2_ (4:1) followed by microwave (MW) heating (600 W).	6 M KOH	52% at 20 A/g	1%	1552	188
Glucose-derived graphene-based AC [174]	NH_4_Cl mix (1:1), heating at 400 °C, heating at 1100 °C, KOH (13:1) activ. at 800 °C.	EMIM-TFSI/AN	90% after 10,000 cycles	Large micro and mesopore presence	3657	175
AC from bacterial cellulose ^2^ [182]	Freezing (liquid N_2_), heat at 900 °C, and KOH (1:1) activ. at 900 °C	6 M KOH	Over 90% after 10,000 cycles	32%	491	167
Porous carbon from starch ^4^ [39]	Graphite addition (20% *w*/*w*), MW heating (140 °C) and pyrolysis at 800 °C	2 M H_2_SO_4_	85% after 10,000 cycles	38%	337	157
Porous carbon from bamboo ^3^ [183]	Mix with KHCO_3_ (4:1) and carbonization at 400 °C	6 M KOH	98.4 after 10,000 cycles at 10 A/g	56%	1425	143
Cashew nut husk derived AC [184]	Heating at 600 °C and KOH (4:1) activ. at 850 °C	6 M KOH	Close to 100% after 4000 cycles	Mainly micro and small mesopores	2742	125/305 *

^1^ Specific capacitance measured at 1 A/g (unless specified otherwise) on a two-electrode system. ^2^ Prepared as a freeze-dried aerogel. ^3^ Using leaving method with KHCO_3_ activation. ^4^ Following a gelation method. * Values in a 3-electrode setup. ** Activ. = Activation.

### 3.4. Hydrothermally Pretreated, Biomass-Derived Electrode Materials for Supercapacitor Applications

As mentioned earlier, materials such as CNTs, graphene, and conductive polymers, have become standard for SC electrodes, demonstrating high capacitances—some even exceeding 800 F/g [185,186]—and retaining over 90% of their capacity after more than 5000 cycles [185]. However, the sustainability and cost concerns linked to these materials necessitate the exploration of alternative options. Consequently, environmentally friendly methods for synthesizing materials derived from biomass are gaining a foothold as viable and potential substitutes. 

Biomass-derived electrodes can be categorized into two groups: those made from biopolymers, which are components of biomass, and those from raw biomass sources, such as wood, bamboo, straw, and hemp. SCs with electrodes from the first group are common, and their synthesis typically involves processes like leavening or gelation, as shown in examples in Table 1. However, HTC of biomass has emerged as a viable method, yielding comparable results in terms of capacitance and cyclability. Table 2 presents examples of electrodes derived from hydrothermally treated biomass.

***Electrodes derived from biopolymers.***

Examples of only the most important types are described.

Cellulose: A comparison between HTC processed cellulose (Table 2) and other synthetic procedures reveals that the HTC process demonstrates potential to compete with treatments like leavening or gelation. For instance, cellulose after leavening and gelation treatments achieves a capacitance of 174 F/g (6 M KOH; 1 A/g) and 205 F/g (1 M H_2_SO_4_; 1 A/g; 3-electrode setup), respectively [183,187], whereas for HTC-treated cellulose very competitive values of 225–150 F/g were reported [132,188,189]. Moreover, HTC-treated cellulose [132] shows a higher surface area of >2400 m^2^/g compared to 1893 and 1364 m^2^/g, respectively, overcoming the disadvantage of not presenting hierarchical 3D porous structures, unlike those obtained by these non-hydrothermal methods, albeit the choice of electrolyte (aqueous vs. ionic liquid) has an important impact on the results. Recent studies on HTC-processed doped cellulose materials and hybrids have demonstrated high capacitance values, albeit in 3-electrode setups. For example, thiourea-doped activated carbon from cellulose presented a high specific surface area of 952.27 m^2^/g and a capacitance of 224 F/g at a current density of 1 A/g [189], and a capacitance value of 208 F/g was recently reported for cellulose nanofiber-based hybrids with GO and AC derived from wheat straw [188]. However, caution must be exercised when comparing these results, as capacitance values obtained from 3-electrode tend to be higher than those from symmetrical supercapacitors. The discrepancy arises because 3-electrode setups measure the capacitance of one half of the cell, while 2-electrode setups measure the whole cell [190].

Starch: When gelated, freeze-dried and carbonized it can yield a product with a surface area of 337 m^2^/g (predominantly mesoporous) and a capacitance of 157 F/g using 2 M H_2_SO_4_ as the electrolyte [39]. In Wei’s study [132], HTC processed starch shows similar capacitance, even though it presents a significantly higher surface area of 2273 m^2^/g, which is predominantly microporous. Furthermore, other studies show that these properties can be improved by doping with other particles, such as ammonium iron (II) sulfate (NH_4_)_2_Fe(SO_4_)_2_, achieving a capacitance of 212 F/g at 1 A/g in a 3-electrode system, mainly due to its high microporosity, representing more than 75% of the total pore volume [191].

**Table 2:** Electrodes prepared from hydrothermally treated biopolymers and raw biomass.

Electrode Material	Synthesis Method	Electrolyte	Stability	Microporosity (%)	BET Area (m^2^/g)	Cap (F/g) ^1^
**Biopolymers**						
AC from chitosan [192]	HTC (250 °C, 4 h), KHCO_3_ activ. ** (750 °C)	1 M H_2_SO_4_	75% at 10 A/g	36%	2124	265/326 *
AC from cellulose and thiourea [189]	HTC (240 °C, 1 h) and KOH (3:1) activ. at 800 °C	6 M KOH	Stable after 20,000 cycles	Mainly microporous	952	224/236 *
Cellulose/AC/GO hydrogel [188]	Straw heating (500 °C) and KOH (3:1) activ. at 700 °C. HTC (180 °C, 1 h) of a cellulose, AC and GO mixture.	Lignin hydrogel	88% after 10,000 cycles	Hierarchical structure (micro, meso and macro)	762	208/565 *
Cellulose-based AC. [132]	HTC (250 °C cell; 230 °C starch, 2 h) and KOH (4:1) activ. at 700 °C	1 M TEABF_4_/AN	65% at 20 A/g.	87%	2457	170
AC from starch. [132]			65% at 20 A/g.	87%	2273	161
Lignin derived AC [193]	HTC (220 °C, 14 h) in H_2_SO_4_ (aq.), KOH (1:1) activ. at 800 °C	6 M KOH	98% after 5000 cycles	76%	1337	110/255 *
**Raw biomass**						
Wood sawdust derived AC. [194]	HTC (120 °C, 2 h) in KOH (aq.) and carbonization at 800 °C	6 M KOH	99% after 5000 cycles.	74%.	1185	244/302 *
AC from coconut shells. [134]	HTC (200 °C, 20 min) in H_2_O_2_ aq., HTC (275 °C, 12 h) in ZnCl_2_ aq. and CO_2_ activ. (800 °C)	0.5 M H_2_SO_4_	88% after 2000 cycles.	Mesoporous structure	2440	207
AC from wood sawdust. [132]	HTC (250 °C, 2 h) and KOH (4:1) activ. at 800 °C	1 M TEABF_4_/AN	75% at 20 A/g.	89%	2967	197
Enteromorpha Prolifera-based AC. [195]	HTC (180 °C, 24 h), heating at 450 °C, KOH (2:1) activ. at 700 °C	6 M KOH	90% after 10,000 cycles.	88%	1528	192
AC from Spirulina platensis and glucose. [133]	HTC (180 °C, 24 h) and KOH (2:1) activ. at 700 °C	6 M LiCl	98% after 10,000 cycles.	93%	2130	177
Jatropha derived AC. [196]	HTC (190 °C, 2 h) and KOH (1:1) activ. at 800 °C	1 M KOH	19% Increase after 5000 cycles.	Large micro and macropores.	747	175
AC from hemp fibers. [131]	HTC (180 °C, 24 h) in H_2_SO_4_ (aq.), KOH (1:1) activ. at 750 °C	BMPY TFSI	90% at 100 A/g	47%	2287	160
AC from corn straws. [136]	HTC (220 °C, 12 h) and KOH (1:1) activ. at 800 °C	6 M KOH	83% after 2000 cycles.	Mainly microporous.	1229	66/271 *

^1^ Specific capacitance measured at 1 A/g (unless specified otherwise) on a two-electrode system. * Values in a 3-electrode setup. ** Activ. = Activation.

Lignin and chitosan: Both emerge as interesting alternatives. Chitosan, in particular, with a similar straight-chain molecular structure to that of cellulose, the main difference being the presence of primary amine groups replacing hydroxyl groups at the C-2 position, demonstrates, after activation, a high capacitance of 265 F/g and good cyclability due to pseudocapacitive processes from O and N-atom doping, combined with even pore distribution and excellent structural properties [192]. Activated lignin [193], whilst having a pseudocapacitive contribution, displays a considerably lower capacitance (110 F/g), see Table 2. This result may be due to different pore distributions between both materials, since lignin is a highly crosslinked polymer and shows a much lower surface area and a less significant mesopore contribution.

It is important to note that all compared materials underwent chemical activation through the addition of KHCO_3_ or KOH. Nevertheless, the KOH:carbon ratios varied among the samples; specifically, in the sample with the lowest microporosity, chitosan, a 1:1 ratio was used, whereas in the other carbon samples a 4:1 ratio was employed. This variation highlights once again the importance of the relationship between the concentration of activating agent and the microporosity of the resulting activated carbon.

***Electrodes derived from raw biomass.***

Electrodes derived from raw biomass often exhibit notable variations in performance characteristics. However, in many cases these electrodes achieve acceptable capacitance despite their relatively modest textural properties. This is primarily attributed to pseudocapacitance generated by N and/or O atoms, as observed for example in materials derived from *Enteromorpha prolifera* [195] or *Spirulina platensis* [133]. In some instances, impressive electrochemical results are obtained as a consequence of using specific reagents to improve the final product. An example of this is found with coconut shells [134], which were synthesized using H_2_O_2_ and ZnCl_2_ in an aqueous medium for the HTC process. This approach yielded a material with good capacitance (207 F/g) and textural properties. Another example is found in Yang’s work [194], where a KOH-assisted HTC process resulted in a material with an excellent capacitance of 244 F/g, which was mainly attributed to heteroatom doping, either from KOH activation or due to the inherent properties of the wood used.

These examples highlight the significance of various synthetic parameters and their direct impact on the end results. For instance, in Wei’s study [132] wood sawdust was heated at 250 °C and then activated using KOH in a 4:1 ratio, while Yang [194] dissolved the raw material in a 5% KOH solution, heated the mixture at 120 °C for varying durations, followed by carbonization, achieving marginally higher capacitance and superior stability, Table 2. A major contributing factor to these outcomes may be the KOH dosage, as discussed previously. Higher KOH dosages are often associated with the development of a more microporous structure, as indicated by increased micropore volume and BET surface area. While this can negatively impact electrochemical performance at higher currents, it may lead to higher specific capacitance values at lower current densities, depending on the type of electrolyte used. 

A comparison between conventional carbonization and HTC processes provide important insights, as the study on jatropha oilcake shows [196]. Electrodes developed from both processes generated materials with capacitances of 145 and 175 F/g, respectively, showing the effectiveness of the HTC material due to its superior structural properties. The higher surface area of the HTC electrode (746 m^2^/g vs. 678 m^2^/g in the conventional process) and the marginally higher pore volume with a slightly smaller pore diameter (1.31 nm vs. 1.77 nm) was observed for HTC, indicating a tendency towards a more microporous product with a larger surface area. This difference in pore size and the other textural properties must be attributed to the different carbonization procedures, since both samples followed the same activation process. Thus, HTC appears to be a more efficient procedure to generate porous materials prior to activation. Furthermore, the author noted that the HTC process was responsible for the formation of smaller sized and highly porous final products, due to disintegration of the feedstock into smaller particles in the aqueous medium during the process, most likely contributing to the observed differences in capacitance between the two samples.

These hydrothermally treated materials hold significant potential for further improvements in electrochemical performance through the incorporation of conductive nanoparticles into the internal structure of the hydrochar. As previously mentioned, several excellent studies have already described the inclusion of nanoparticles, such as GO, CNT, metal oxides, and MXenes, into the carbonaceous structure of hydrochar to enhance electrochemical properties [188,197,198,199]. However, reports on the combination of TMDCs with hydrothermally treated biomass for energy storage applications in batteries and supercapacitors are less common. The Section 3.5 of this review specifically addresses the incorporation of this family of nanoparticles.

### 3.5. TMDCs: Energy Storage and Other Promising Applications, Enhancing Biomass-Derived Materials

There are over 40 different TMDC types, falling into the categories of metals (e.g., TiS_2_ and VSe_2_), superconductors (e.g., TaS_2_ and NbS_2_), semi-metals (e.g., MoTe_2_ and WTe_2_), and semiconductors (MoS_2_, MoSe_2_, WS_2_, and WSe_2_). TMDCs exhibit interesting band structures with tunable bandgaps, a crucial factor in determining the properties and applications of 2D materials. Among the transition metal oxides, tungsten trioxide (WO_3_) stands out due to its high intrinsic density, high mechanical stability, and favorable electrochemical redox characteristics [200].

A fundamental advantage of TMDC nanostructures over carbon or metal oxide nanoparticle equivalents is their low toxicity and biocompatibility, enabling their use even for medical applications [55]. Research has shown that TMDCs have excellent performance in biosensing and bioimaging that has led to TMDC platform technologies for medical diagnosis [201]. These include electrochemical, fluorescent, chemiluminescent, colorimetric, thermal, field effect transistor and piezoelectric crystal biosensors, which can be used for quantitative detection of biological substances to very low concentrations, and bioimaging through fluorescence imaging, computed tomography, magnetic resonance imaging, photoacoustic imaging, and multimodal imaging [201]. However, surface modification is necessary to further improve TMDCs properties in these fields. Essentially, 2D TMDCs are chemically inert, conferring the advantage of structural stability along with inherent difficulties for their effective surface functionalization. On the other hand, the unique properties of TMDCs have been the main driving force in the development of pollution reduction applications. Zhang et al. [202]. present a view of the state-of-the-art in applications of various TMDCs in pollution mitigation, including gas adsorption and removal, gas sensing, wastewater treatment, flue cleaning, and CO_2_ valorization and conversion, highlighting the growing potential of TMDCs in environmental safety.

The combination of TMDCs with HTC biomass-derived materials represents a novel direction in electrochemical energy storage, particularly for their application as electrodes in batteries and supercapacitors. While extensive literature exists on TMDC/HTC in Li-ion batteries, research in SCs is less prevalent. Table 3 compares a series of TMDC-containing HTC-processed carbonaceous materials and their reported electrochemical performance in batteries and supercapacitors.

In the realm of Li-ion batteries, a noteworthy study involves the hydrothermal treatment of glucose followed by mixing with TMDCs in an additional HTC step. The method produces a MoS_2_/HTC electrode for a battery, which achieves a stable capacity of 484 mA·h/g using a 1 M LiPF_6_ solution in a mixture of ethyl carbonate (EC), ethyl methyl carbonate (EMC), and diethyl carbonate (DEC) as the electrolyte. Remarkably, it retains a coulombic efficiency of 98% after 50 charge/discharge cycles [207]. Zhao’s work [204] also subjected biomass to HTC, previously mixing it with MoS_2_ precursors, obtaining a final carbon/TMDC electrode with MoS_2_ nanoflowers accumulated over the carbon surface. This material was tested for Li-ion batteries and yielded a capacity exceeding 1300 mA·h/g that, after 100 cycles remained over 1100 mA·h/g in a 1 M LiPF_6_ solution, Table 3. The authors attribute these results to a successful intercalation of Li ions between the MoS_2_ layers, as well as the superior conductivity provided by the carbon, significantly improving MoS_2_ conductivity. Furthermore, these authors conducted various electrochemical experiments with samples prepared at different residence times, finding that a sample prepared at 1 h displayed more active sites and better textural properties, leading to a superior electrochemical performance.

Additionally, the combined incorporation of graphitic particles has been reported, Table 3. For example, the HTC treatment of chitosan and graphene oxide, followed by a facile synthesis, led to MoS_2_/graphene structures with a capacity exceeding 1000 mA·h/g, when used as electrodes in a Li-ion battery with 1 M LiPF_6_ solution in a mixture of EC and DMC as electrolyte. This capacity remained stable over 100 charge/discharge cycles. The authors credit this stability to the enhanced surface area for electrolyte interaction, improved pathways for lithium ion insertion and extraction, and condensed routes for swift charge carrier diffusion [205]. These results are significant, especially when compared to other hydrothermal processes incorporating TMDCs, but not biomass derived products. For instance, hydrothermally prepared MoO_2_/MWCNTs electrodes exceed a capacity of 1200 mA·h/g, dropping to 1143 after 200 cycles [206]. Similarly, microwave-assisted HTC of MoS_2_/Graphene electrodes achieve more than 1300 mA·h/g, with a capacity of 1127 after 100 cycles [203].

In the field of electrochemical capacitors, some noteworthy studies have been conducted on the hydrothermal treatment of carbonaceous materials incorporating TMDCs and combined with other nanoparticles. For example, a study involving reduced graphene oxide (rGO), produced via HTC of graphene oxide, mixed with WS_2_ sheets through a subsequent hydrothermal process, yielded electrodes capable of achieving nearly 275 F/g (measured at 1 A/g) in a two-electrode supercapacitor immersed in 1 M Na_2_SO_4_ solution. Remarkably, these materials retained up to 94% of their original capacitance after 1000 cycles, Table 3. This performance is attributed to the high electrical conductivity and homogeneous coverage of the thin-layered TMDC and rGO sheets, facilitating electrolyte ion transport [208]. A similar approach was followed by Huang, who used hydrothermal treatment of MoS_2_ precursors and graphene oxide to produce electrode materials that exhibited a capacitance of 243 F/g (1 M Na_2_SO_4_; 3-electrode setup) with a capacitance retention of 92% after 1000 cycles. The authors attribute these findings to three primary factors: (i) the coating of TMDCs on graphene nanosheets enhances pore formation, thereby facilitating ion transport; (ii) the substantial surface area of the materials result in a reduced diffusion length for ion transfer, reaching 103 m^2^/g compared to the original 48 m^2^/g of graphene; and (iii) the enhanced conductivity provided by graphene [212]. In another example, Gao produced MoS_2_/carbon composites via supramolecular self-assembly using β-cyclodextrins and L-cysteine [211] that yielded a capacitance of 394 F/g (5 mV/s) in a 1 M Na_2_SO_4_ solution through a hydrothermal process. While the authors explain this result by the presence of carbon inhibiting the restacking of TMDC layers and producing an almost 5-fold increase in surface area compared to that of pure MoS_2_, the resulting surface area is still very low when compared to other systems. The most likely explanation lies in the synergistic effects of the combination of nanoparticles that demonstrate an improvement in conductivity and good disposition of electrochemically active sites provided by MoS_2_.

Following a related strategy, Xing et al. prepared WO_3_ nanoparticles and dispersed them onto rGO using a hydrothermal technique, obtaining a significantly higher specific capacitance of 580 F/g than that of pure WO_3_ (255 F/g) at 1 A/g in 2 M KOH [210]. The improved capacitance of rGO/WO_3_ composites they related to a 70% increase in specific surface area (approximately 17 m^2^/g in the final material) and excellent electrical conductivity, promoting ionic diffusion and charge transfer kinetics. Further, Liu at al. [218] successfully grew WO_3_ nanowires on graphene sheets employing a seed-mediated hydrothermal method using a negative electrode. The important improvement obtained in specific capacitance (800 mF/cm^2^ at current density of 1 mA/cm^2^) was clearly due to synergistic effects between graphene and WO_3_ nanowires. Similarly, tungsten disulfide (WS_2_)/active carbon fiber composites were prepared using electrospinning, carbonization, and subsequent hydrothermal methods [209]. These nanocomposites demonstrate a high capacitance of 255 F/g in a two-electrode setup at a current density of 1 A/g in a 1 M KOH electrolyte, which increased to 600 F/g in a 3-electrode configuration. The authors attribute the improvements to the thin WS_2_ nanosheets and high conductivity of the activated carbon, which also led to a three-fold increase in surface area (10.8 m^2^/g compared to 3.7 m^2^/g for pure WS_2_). This highlights the critical role of high conductivity and the presence of active sites, both of which are essential for mitigating other performance-related challenges, such as limited surface area.

Focusing on hydrothermally treated biomass, Hu’s study [217], demonstrated that the HTC of glucose mixed with anodic aluminum oxide as a template and a MoS_2_ precursor resulted in electrodes with a capacitance of 210 F/g, which increased by 5% after 1000 cycles. The pore structure was found to be a crucial role factor in the electrochemical performance of these composites. The authors suggest that hydrothermal treatment leads to the carbothermal reduction in MoS_3_, forming MoS_2_ and simultaneously generating tubular pores within the carbonaceous framework. These pores, along with the enhanced conductivity provided by the carbon, significantly contribute to the observed capacitance values. This underscores the critical importance of pore structure and the optimal modification of hydrothermal treatment parameters that influence the structure in the development of effective electrodes. Another study using glucose [214] followed a similar HTC process, blending polyethylene glycol PEG, thiourea, MoS_2_ precursor, and glucose to develop electrodes for a two-electrode supercapacitor. These materials achieved a capacitance of 160 F/g (measured at 0.86 A/g with a 1 M Na_2_SO_4_ electrolyte) and retained 95% of their original capacitance after 1000 charge/discharge cycles. As previously discussed, the nature of the pore structure is crucial in explaining these results. The authors attribute the development of flower-like MoS_2_ mesoporous structures to the creation of pathways that facilitate rapid ion diffusion, which explains the relatively low specific surface area of only 69 m^2^/g. The methodology employed produced a material with a high interfacial area between the electrode and electrolyte. Additionally, the close contact between TMDCs and hydrochar contributed to increased conductivity in the final composite, thereby improving its overall performance. In a related study, Zhao prepared carbonaceous materials via the HTC of corn stalk with MoS_2_ precursors [204]. These materials, which found application in Li-ion batteries as previously described, also proved very effective in supercapacitors, achieving superior specific capacitance values of 338 F/g with a stability of nearly 80% after 5000 cycles.

Several alternative approaches have also been developed to produce high-performing electrodes for supercapacitors from biomass. For example, Sangeetha [213] first developed activated carbons from tendu leaves through conventional carbonization and chemical activation. These were then subjected to HTC to create structural defects and subsequently mixed with hydrothermally developed MoS_2_. This method yielded a highly porous carbonaceous material with a surface area exceeding 1500 m^2^/g that contained a combination of micro and mesopores. These features led to high capacitance values of 261 F/g (at 2 mV/s), with excellent stability over 5000 cycles. Moreover, this material also found application in the hydrogen evolution reaction (HER). A similar approach was undertaken by Wang [215], who first carbonized corncob at 750 °C, then mixed the material with MoS_2_ precursors, followed by hydrothermal treatment and chemical activation with KOH. This procedure yielded predominantly microporous materials with a small contribution of mesopores. These materials exhibited a capacitance of 333 F/g measured in a 3-electrode setup, but only 38 F/g in a symmetrical supercapacitor. Nevertheless, this carbonaceous electrode demonstrated excellent stability, retaining more than 90% of its original capacitance after 2000 cycles and 82% after 7000 cycles. This stability is attributed to the conductivity enhancement provided by the carbon, the increased number of active sites for charge transfer, and the enlarged surface area in contact with the electrolyte, which collectively shorten the charge/discharge cycles. Additionally, Lin’s work [216] developed a porous carbon material via conventional carbonization and chemical activation. This material was subsequently mixed with MoS_2_ precursors and subjected to HTC, resulting in a predominantly mesoporous material with a surface area of 320 m^2^/g. The presence of carbon not only increased conductivity, but also prevented TMDC agglomeration, thereby increasing active sites and providing larger ion diffusion pathways. The synergistic effect of these materials led to excellent electrochemical performance, with a capacitance of 361 F/g (in a 3-electrode setup) and a 94% capacitance retention after 2000 cycles. 

In summary, the diverse methods for incorporating TMDCs into biomass-derived hydrochars emphasize the need to tailor each step to achieve optimal electrochemical performance. Feedstock selection clearly plays a crucial role in determining the properties of the resulting materials, as its properties directly influence surface area, pore structure, nanoparticle distribution and conductivity. Adjusting activation techniques, such as activation type and degree, can significantly enhance surface area and create hierarchical pore structures, facilitating electrolyte accessibility and ion transport—key factors for high-performance supercapacitors. Additionally, the synergy between TMDCs and biomass-derived carbon materials can further boost conductivity and refine the textural architecture, increasing the number of active sites available for charge transfer. The combination of these diverse strategies highlights the potential of TMDC–biomass hybrids as efficient and versatile supercapacitor electrodes.

## 4. Concluding Remarks and Future Perspectives

This review has examined the role of hydrothermal carbonization in synthesizing effective electrode materials for supercapacitors. Key parameters such as temperature and residence time were identified as fundamental factors influencing the resulting carbon structure. Specifically, for biomass-derived electrode materials, temperatures of approximately 200 °C and residence times of 1 to 4 h were shown to be optimal for producing carbons with enhanced porosity after activation, leading to improved capacitance and cycling stability. Notably, the presence or inclusion of heteroatoms, particularly oxygen and nitrogen functional groups, also positively impacts efficiency of the HTC process. The impact of other variables, such as hydrothermal reagents, and activation parameters, on the final materials’ electrochemical behavior, was also discussed.

While the enormous diversity of biomass sources may in principle present a complicated challenge, the numerous benefits of the HTC process contribute to its growing use in preparing sustainable carbons and producing value-added materials from almost any type of biomass that can be employed for energy storage devices. This expansion includes innovative modifications of the original methods, aimed at enhancing both the properties and electrochemical performance of the resulting products. These include post-activation, the introduction of doping agents, heteroatoms, and the incorporation of diverse nanoparticles including TMDCs. 

Many of these developments have yielded promising results positioning HTC biomass-derived materials as viable alternatives to non-renewable counterparts in electrochemical energy storage devices. Notably, capacitance values exceeding 300 F/g have already been achieved with more than 90% capacitance retention after 1000 cycles. However, in global terms, challenges remain, particularly in improving energy density and capacitance at high charge/discharge currents, possibly due to residual oxygenated functional groups affecting conductivity. Despite these challenges, overall performance metrics, including power density, stability, and capacitance, are comparable or even superior to those of other carbons prepared without HTC pretreatment or those derived from fossil fuels.

Finally, the emergence of novel approaches, such as the development of HTC biomass-derived TMDC hybrids, opens up new possibilities for addressing remaining limitations. Individually, these nanoparticles have shown remarkable electrochemical performance attributed to an improvement in the contact area between electrode and electrolyte, enhanced conductivity and the availability of active sites conducive to pseudocapacitance. Their biocompatibility and reduced environmental impact compared to other carbon-based nanoparticles further position them as excellent candidates for their incorporation into HTC-derived carbon materials. The diversity of results obtained thus far reveals a wealth of opportunities, not only in electrochemical energy storage but also in other high-value applications related to environmental sustainability, biosensing and bioimaging for medical diagnostics.

## Figures and Tables

**Table 3:** Representative examples of the electrochemical performance of hydrothermally treated carbonaceous, TMDC-containing electrodes.

	**Li-Ion Batteries**				
**Electrode Material**	**Synthesis Method**	**Electrolyte ^1^**	**Stability**		**Capacity (mA·h/g)**
MoS_2_/Graphene [203]	HTC (180 °C,12 h) of MoS_2_ precursors and graphene	1 M LiPF_6_ solution in an EC/DEC mixture	1127 mA·h/g after 200 cycles		>1300
Cornstalk-derived C/MoS_2_ [204]	HTC (200 °C, 1 h) of precursors and corn; pyrolysis at 1000 °C	1 M LiPF_6_ solution in a mixture of EC/DEC/DMC	1129 mA·h/g after 200 cycles		> 1300
AC from chitosan/graphene oxide/MoS_2_ [205]	HTC (240 °C, 24 h) of all materials and annealing at 800 °C	1 M LiPF_6_ solution in an EC/DMC mixture	Stable over 100 cycles		>1000
MoO_2_/Multiwalled carbon nanotubes [206]	HTC (200 °C, 36 h) of CNT and MoO_2_ precursors.	1 M LiPF_6_ solution in an EC/DMC mixture	1143 mA·h/g after 200 cycles		>1200
AC from glucose and MoS_2_ [207]	HTC (200 °C, 48 h) of glucose; HTC (200 °C, 18 h) of MoS_2_ precursor and hydrochar. Pyrolysis at 600 °C	1 M LiPF_6_ solution in an EC/EMC/DEC mixture	98% retention after 50 cycles		484
	**Supercapacitors**				
**Electrode material**	**Synthesis method**	**Electrolyte**	**BET area (m^2^/g)**	**Stability**	**Capacitance ^2^ (F/g)**
Graphene oxide/WS_2_ [208]	HTC (265 °C, 24 h) of GO and WS_2_ precursors	1 M Na_2_SO_4_	-	94% after 1000 cycles	274 *
AC fiber/WS_2_ [209]	Fiber activ. *** (800 °C) with KOH (3:1). HTC (180 °C, 24 h) of AC and WS_2_ precursors	1 M KOH	11	93% after 1000 cycles	255 */600 **
Graphene oxide/WO_3_ [210]	Hydrothermal heating (90 °C, 3 h) of precursor; heating at 500 °C. HTC (180 °C, 12 h) of WO_3_/GO	2 M KOH	17	>320 F/g after 1000 cycles	580 **
Carbon/MoS_2_ [211]	HTC (200 °C, 12 h) of all precursors	1 M Na_2_SO_4_	16	60% after 2000 cycles	394 ** at 5 mV/s
Graphene/MoS_2_ [212]	HTC (180 °C, 36 h) of GO and MoS_2_ precursor.	1 M Na_2_SO_4_	103	92% after 1000 cycles	243 **
**Biomass-derived electrodes for supercapacitors**					
Tendu leaf-derived AC/MoS_2_ [213]	Heating (450 °C) and KOH (3:1) activ. (650 °C) of leaves. HTC (180 °C, 20 h) of MoS_2_ precursor; HTC (180 °C,12 h) and heating (800 °C) of AC	1 M Na_2_SO_4_	1509	89% after 5000 cycles	261 * at 2 mV/s
Glucose/PEG/Thiourea/MoS_2_ [214]	HTC (200 °C, 24 h) of MoS_2_ precursor and rest of materials.	1 M Na_2_SO_4_	69	95% after 1000 cycles	186 *
Corncob-derived carbon/MoS_2_ [215]	Pyrolysis (750 °C) of corncob. HTC (200 °C, 16 h) of MoS2 precursors and carbon. Mix with KOH and drying.	1 M Na_2_SO_4_	101	82% after 7000 cycles	38 */333 **
Pomelo peel-derived AC/MoS_2_ [216]	KOH (5 mol/L) activ. (700 °C) of biomass. HTC (220 °C, 24 h) of carbon and MoS_2_ precursor	3 M KOH	320	94% after 2000 cycles	361 **
Cornstalk-derived C/MoS_2_ [204]	HTC (200 °C, 1 h) of precursors and corn; pyrolysis at 1000 °C	1 M Na_2_SO_4_ in a mixture of EC/DEC/DMC	326	79% after 5000 cycles	338 **
Glucose/Al_2_O_3_/MoS_2_ [217].	HTC (200 °C, 24 h) of glucose and MoS_2_ precursor. Annealing at 500 °C	3 M KOH	-	Increase 5% after 1000 cycles	210 **

^1^ Ethyl carbonate (EC), Ethyl methyl carbonate (EMC), Diethyl carbonate (DEC), Dimethyl carbonate (DMC). ^2^ Specific capacitances measured at 1 A/g unless otherwise stated. * In a 2-electrode setup. ** In a 3-electrode setup. *** Activ. = Activation.

## Data Availability

Data sharing is not applicable.
